# Association between diuretics and successful discontinuation of continuous renal replacement therapy in critically ill patients with acute kidney injury

**DOI:** 10.1186/s13054-018-2192-9

**Published:** 2018-10-10

**Authors:** Junseok Jeon, Do Hee Kim, Song In Baeg, Eun Jeong Lee, Chi Ryang Chung, Kyeongman Jeon, Jung Eun Lee, Wooseong Huh, Gee Young Suh, Yoon-Goo Kim, Dae Joong Kim, Ha Young Oh, Hye Ryoun Jang

**Affiliations:** 1 Division of Nephrology, Department of Medicine, Samsung Medical Center, Sungkyunkwan University School of Medicine, Seoul, Republic of Korea; 2 Department of Critical Care Medicine, Samsung Medical Center, Sungkyunkwan University School of Medicine, Seoul, Republic of Korea

**Keywords:** Acute kidney injury, Continuous renal replacement therapy, Diuretics

## Abstract

**Background:**

Despite aggressive application of continuous renal replacement therapy (CRRT) in critically ill patients with acute kidney injury (AKI), there is no consensus on diuretic therapy when discontinuation of CRRT is attempted. The effect of diuretics on discontinuation of CRRT in critically ill patients was evaluated.

**Methods:**

This retrospective cohort study enrolled 1176 adult patients who survived for more than 3 days after discontinuing CRRT between 2009 and 2014. Patients were categorized depending on the re-initiation of renal replacement therapy within 3 days after discontinuing CRRT or use of diuretics. Changes in urine output (UO) and renal function after discontinuing CRRT were outcomes. Predictive factors for successful discontinuation of CRRT were also analyzed.

**Results:**

The CRRT discontinuation group had a shorter duration of CRRT, more frequent use of diuretics after discontinuing CRRT, and greater UO on the day before CRRT discontinuation [day minus 1 (day − 1)]. The diuretics group had greater increases in UO and serum creatinine elevation after discontinuing CRRT. In the CRRT discontinuation group, continuous infusion of furosemide tended to increase UO more effectively. Multivariable regression analysis identified high day − 1 UO and use of diuretics as significant predictors of successful discontinuation of CRRT. Day − 1 UO of 125 mL/day was the cutoff value for predicting successful discontinuation of CRRT in oliguric patients treated with diuretics following CRRT.

**Conclusions:**

Day − 1 UO and aggressive diuretic therapy were associated with successful CRRT discontinuation. Diuretic therapy may be helpful when attempting CRRT discontinuation in critically ill patients with AKI, by inducing a favorable fluid balance, especially in oliguric patients.

## Background

Acute kidney injury (AKI) is a major morbidity in critically ill patients and is associated with high mortality [1]. The overall incidence of AKI in critically ill patients is 6–20% and varies depending on the patient’s medical status [2–4]. Major complications of AKI such as volume overload, severe metabolic acidosis or hyperkalemia, and overt uremic signs are well-known indications for renal replacement therapy (RRT) [5, 6]. Continuous renal replacement therapy (CRRT) is the preferred treatment option for critically ill patients with AKI requiring RRT due to better hemodynamic tolerance and steadier solute control [7–9].

Despite aggressive application of CRRT in critically-ill patients with AKI over the last decade, evidence-based guidelines for optimal timing and adequate methods for CRRT discontinuation are lacking [10]. Assessment of AKI and predicting the prognosis of renal recovery after discontinuing CRRT remain challenging. Several studies have reported lower sequential organ failure assessment scores, fewer prior CRRT cycles, younger age, and higher urine output (UO) after discontinuing CRRT as predictors of renal recovery after AKI requiring CRRT [11–13].

UO is partly manageable by appropriate administration of diuretics in patients with AKI with volume overload [14]. A recent study reported that furosemide was not associated with more frequent renal recovery despite increasing urine volume [15], whereas a previous meta-analysis showed that loop diuretics were associated with shorter duration of RRT [16]. Despite great feasibility and possible cost-effectiveness, contradictory studies on the clinical effectiveness of diuretics after CRRT have prevented a consensus on diuretic therapy, even in patients with AKI at risk of volume overload when CRRT is discontinued. We hypothesized that diuretics may facilitate successful discontinuation of CRRT. In this study, the effects of diuretics on the clinical course of critically ill patients with AKI were investigated, focusing on UO and recovery of renal function following CRRT.

## Methods

### Study design and patient selection

**Fig. 1:**
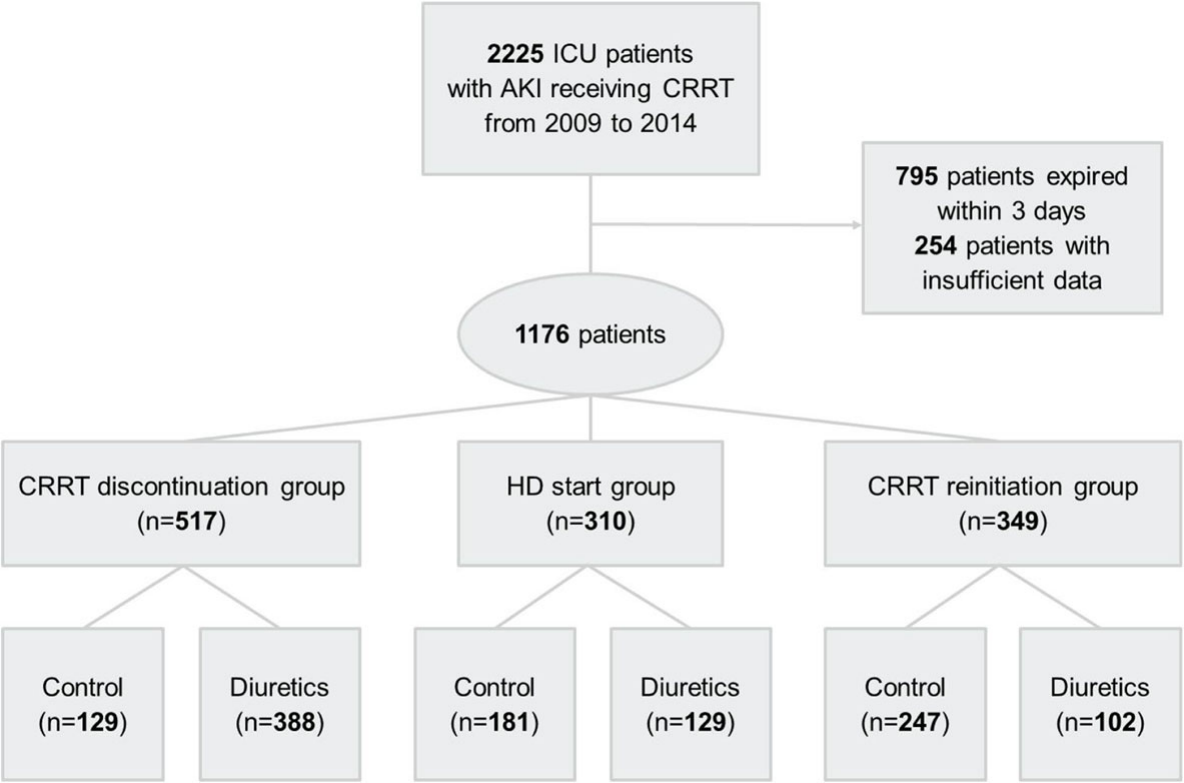
Study design. From September 2009 to December 2014, 2225 ICU patients treated with continuous renal replacement therapy (CRRT) in whom CRRT discontinuation was attempted were screened. A total of 1176 patients were included and classified into three groups based on discontinuation of CRRT. Patients were further divided into control or diuretics groups depending on administration of diuretics after discontinuation of CRRT. The control group included patients who were not treated with diuretics. AKI, acute kidney injury; HD, hemodialysis; ICU, intensive care unit

A total of 2225 adult ICU patients (≥ 18 years old), who received CRRT for AKI and in whom discontinuation of CRRT was attempted between September 2009 and December 2014, were screened at Samsung Medical Center. This retrospective cohort study eventually included 1176 patients after excluding patients who died within 3 days after CRRT discontinuation (*n* = 795) and patients with dialysis-dependent end-stage renal disease (ESRD) or insufficient data (*n* = 254). All patients were categorized into three groups depending on re-initiation of RRT within 3 days after discontinuing CRRT: the CRRT discontinuation (*n* = 517), hemodialysis (HD) initiation (*n* = 310), and CRRT re-initiation (*n* = 349) groups. All three groups were further categorized depending on diuretic use following CRRT. Patients for whom diuretics were not prescribed were defined as the control group (Fig. 1). Changes in renal function and UO during the 3 days following CRRT were compared. The Institutional Review Board (IRB) of Samsung Medical Center approved the study protocol in compliance with the Declaration of Helsinki, and informed consent was waived because of the retrospective and non-interventional design of the study (IRB number 201510110).

### Clinical assessment and outcome measures

Clinical data including age, sex, CRRT duration, blood pressure, vasopressor requirement, UO, and underlying comorbidities were extracted from electronic medical records. Laboratory data including blood urea nitrogen (BUN) and serum creatinine level (sCr) were also collected. The estimated glomerular filtration rate (eGFR) was based on a modified Modification of Diet in Renal Disease (MDRD) equation using sCr [17]. Baseline data were collected on the day that CRRT was initiated and 1 day before cessation of CRRT. AKI was defined as the presence of at least one of the following criteria based on the Kidney Disease: Improving Global Outcomes (KDIGO) clinical practice guidelines for AKI: (1) increase in sCr ≥ 0.3 mg/dL within 48 h; (2) increase in sCr to ≥ 1.5 times baseline within the prior 7 days; (3) urine volume < 0.5 mL/kg/h for 6 h [18].

Successful discontinuation of CRRT was defined as no RRT requirement within 3 days after stopping CRRT. Use of diuretics was defined as administration of diuretics after stopping CRRT. Day 0 was defined as the day of attempting to stop CRRT. UO measured by urine volume (milliliters/day) and renal function measured by sCr were serially compared among groups depending on re-initiation of RRT or use of diuretics from 1 day before CRRT discontinuation [day minus 1 (day − 1)] to day 3. To evaluate the effectiveness of diuretics, serial changes in UO and sCr were compared to day − 1 after logarithmic transformation. Changes in UO and sCr at day χ were analyzed using log (day χ UO/day − 1 UO) and log (day χ sCr/day − 1 sCr), respectively, due to the skewed distribution.

Associations between UO, sCr, and the pattern of furosemide administration (continuous intravenous infusion (CIV); intermittent intravenous administration (INT); per oral administration (PO) subgroups) in the CRRT discontinuation group were also analyzed. Patients treated with both furosemide and other diuretics such as spironolactone or thiazides were defined as the combination group. Diuretic treatment including furosemide was aimed at targeting a urine volume ≥ 0.5 mL/kg/h. Combination diuretic treatment or continuous infusion of furosemide was additionally determined depending on the patient’s urine volume, although there was no quantifiable criterion. Furthermore, predictive factors for successful discontinuation of CRRT were investigated.

### Indications and use of CRRT

All patients were managed in a 128-bed mixed medical, cardiac, neurologic, and surgical ICU equipped with 17 CRRT machines and staffed with full-time intensivists. Nephrologists and intensivists discussed and decided when to initiate or discontinue CRRT. We followed the general indication of RRT (risk of volume overload, hyperkalemia, metabolic acidosis, and symptomatic uremia etc.) considering patients’ underlying disease or specific conditions such as heart failure or transfusion requirement. The choice between CRRT and HD was determined by the attending intensivist and nephrologist considering the general CRRT indications and hemodynamic stability. We attempted to discontinue CRRT when the overall clinical status of the patient was stabilized, reaching and maintaining normokalemia and euvolemic/hemodynamically stable status with no significant metabolic acidosis or uremia for a sufficient time period ≥ 48 h. There was no difference in fluid balance between groups at the time of CRRT discontinuation. Intended discontinuation of CRRT was attempted in patients with euvolemic status. The final decision was made by the multidisciplinary critical care team composed of intensivists, attending physicians, and nephrologists regardless of the UO.

Generally, continuous veno-venous hemodiafiltration was performed and the initial target dose was prescribed as 35 mL/kg/h (dialysate flow rate plus replacement flow rate) followed by individual adjustment [19]. Hemosol B0 (Baxter, Deerfield, IL, USA) and Multibic 4 K (Fresenius Medical Care, Bad Homburg, Germany) were used as dialysate or replacement fluids.

### Statistical analyses

Continuous variables were expressed as mean ± standard deviation (SD) (normal distribution) or median with interquartile range (IQR) (non-normal distribution). The significance of differences between groups was determined by the unpaired *t* test or Mann-Whitney test as appropriate. One-way analysis of variance followed by Bonferroni’s correction or the Kruskal-Wallis test was used for comparing more than two groups as appropriate. Multivariable regression analyses with the manual backward stepwise procedure were performed to identify predictive factors for successful discontinuation of CRRT. The predictive ability of UO was assessed using the area under the receiver operating characteristic (ROC) curve method. A *p* value <0.05 was considered statistically significant. All statistical analyses were conducted using IBM SPSS statistics 23 (IBM Corporation, Armonk, NY, USA).

## Results

### Baseline characteristics

The baseline characteristics of patients in the three groups are summarized in Table 1. The mean age, sex, and underlying comorbidities at admission were similar among groups, although the HD initiation group contained a larger proportion of hypertensive patients.

**Table 1:** Baseline characteristics

Clinical variables	CRRT discontinuation group (*n* = 517)	HD initiation group (*n* = 310)	CRRT re-initiation group (*n* = 349)	*p* value
Age (years)	62.1 (15.2)	61.0 (14.9)	61.9 (14.8)	0.613
Male	330 (63.8%)	197 (63.5%)	222 (63.6%)	0.996
Comorbidity				
Hypertension	185 (35.8%)	142 (45.8%)	95 (27.2%)	< 0.001^cde^
Diabetes mellitus	136 (26.3%)	94 (30.3%)	81 (23.2%)	0.118
Ischemic heart disease	36 (7.0%)	17 (5.5%)	17 (4.9%)	0.408
Liver cirrhosis	31 (6.0%)	24 (7.7%)	34 (9.7%)	0.123
Heart failure	22 (4.3%)	19 (6.1%)	15 (4.3%)	0.420
At CRRT initiation				
BUN (mmol/L)	30.0 (12.1)	21.8 (10.8)	19.9 (10.8)	0.106
Serum creatinine (μmol/L)	228.1 (145.0–351.8)	353.6 (223.7–548.1)	225.4 (147.6–337.7)	< 0.001^ac^
Urine output (mL/day)	570 (180–1308)	116 (0–361)	244 (32–835)	< 0.001^abc^
Day −1 before CRRT discontinuation				
BUN (mmol/L)	13.4 (9.1)	13.6 (7.9)	13.4 (7.75)	0.924
Serum creatinine (μmol/L)	134.4 (91.9–209.5)	191.8 (122.9–302.3)	143.2 (99.0–224.5)	< 0.001^ac^
Urine output (mL/day)	565 (252–1250)	45 (5–190)	60 (10–289)	< 0.001^ab^
Mean blood pressure (mmHg)	79.4 (15.1)	78.5 (15.4)	79.6 (15.0)	0.631
Vasopressor use	342 (66.2%)	202 (65.2%)	241 (69.1%)	0.530
Duration of CRRT (days)	3.4 (2.6)	4.8 (5.1)	4.8 (5.0)	< 0.001^ab^
Use of diuretics after CRRT	388 (75.0%)	129 (41.6%)	102 (29.2%)	< 0.001^abc^

Continuous variables following a normal distribution are expressed as mean (standard deviation) or median (interquartile range). Categorical variables are expressed as number (percentage). One-way analysis of variance followed by the Bonferroni correction or the Kruskal-Wallis test followed by the Mann-Whitney test was performed for analysis of the continuous renal replacement therapy (CRRT) discontinuation group versus the hemodialysis (HD) initiation group versus the CRRT re-initiation group. Categorical variables were compared using the χ^2^ test

*Abbreviations: BUN* blood urea nitrogen, *Day − 1* one day before CRRT discontinuation

^a^*p* ≤ 0.001 for CRRT discontinuation group versus HD initiation group

^b^*p* ≤ 0.001 for CRRT discontinuation group versus CRRT re-initiation group

^c^*p* ≤ 0.001 for HD initiation group versus CRRT re-initiation group

^d^*p* ≤ 0.01 for CRRT discontinuation group versus HD initiation group

^e^*p* ≤ 0.01 for CRRT discontinuation group versus CRRT re-initiation group

^f^*p* ≤ 0.01 for HD initiation group versus CRRT re-initiation group

The CRRT discontinuation group had greater UO compared with other groups at 1 day prior to starting CRRT and 1 day prior to stopping CRRT, and had a shorter duration of CRRT compared to other groups (Table 1). In the CRRT discontinuation group, the proportion of patients receiving diuretics after discontinuation of CRRT was greater compared to the other groups. Mean blood pressure and the proportion of patients with vasopressor administration were similar among groups on day − 1.

### Overall changes in UO and renal function

**Fig. 2:**
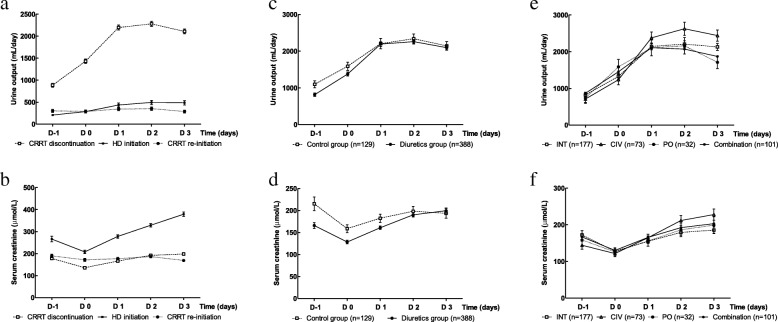
Changes in urine output and renal function after discontinuation of continuous renal replacement therapy (CRRT). **a** Overall changes in urine output. The urine output of the CRRT discontinuation group was significantly increased compared with that of other groups after stopping CRRT. **b** Overall changes in serum creatinine. The hemodialysis (HD) initiation group had significantly elevated serum creatinine compared with other groups. **c** Changes in urine output between the control and the diuretics subgroups in the CRRT discontinuation group. The diuretics subgroup had a significant increment in urine output. **d** Changes in serum creatinine between the control and the diuretics subgroups in the CRRT discontinuation group. The diuretics subgroup had significantly elevated serum creatinine. **e** Changes in urine output depending on method of administration of diuretics. The CIV group showed a tendency to increase urine output more effectively compared to other methods of delivery. **f** Changes in serum creatinine depending on the administration method of diuretics. The continuous intravenous infusion of furosemide (CIV) group had significantly elevated serum creatinine. INT, intermittent intravenous infusion of furosemide; PO, furosemide administration per oral or Levin tube; D, day

Figure 2a and b show serial changes in the UO and sCr of each group. UO was greater and increased significantly in the CRRT discontinuation group compared to other groups (CRRT discontinuation versus HD initiation versus CRRT re-initiation: day 3 UO, median (IQR), mL/day, 1910 (1230~2770) vs. 55 (0~567) vs. 42 (5~250), *p* < 0.001; changes in UO on day 3, median (IQR), 0.49 (0.21~0.81) vs. 0.00 (− 0.48~0.71) vs. − 0.20 (− 0.63~0.33), *p* < 0.001). The HD initiation group experienced a significant increase in sCr after stopping CRRT compared to other groups (CRRT discontinuation versus HD initiation versus CRRT re-initiation: day 3 sCr, median (IQR), μmol/L, 174.2 (106.1~263.4) vs. 345.6 (254.6~477.4) vs. 141.4 (99.0~216.6), *p* < 0.001; changes in sCr on day 3, median (IQR), 0.24 (− 0.19~0.57) vs. 0.54 (0.16~0.92) vs. 0.00 (− 0.35~0.30), *p* < 0.001).

### Effectiveness of diuretics

Patients who were treated with diuretics (*n* = 619) had greater increases in UO and sCr after discontinuation of CRRT compared to patients not treated with diuretics (control, *n* = 557). UO during the 3 days after discontinuation of CRRT was greater in the diuretics group compared to the control group.

In the CRRT discontinuation group, patients who were treated with diuretics had greater increases in UO and sCr elevation after discontinuation of CRRT compared to patients without diuretics (Fig. 2c and d). Both UO and sCr were greater in the control group than the diuretics group on day − 1, whereas median UO and sCr on day 3 were similar between groups (control group versus diuretics group: median (IQR) UO, mL/day, 810 (335~1457.5) vs. 512.5 (235~1088.8) on day − 1, *p* = 0.002, 1870 (1080~2887.5) vs. 1920 (1256.3~2713.8) on day 3, *p* = 0.902; median (IQR) sCr, μmol/L, 148.5 (95.5~280.7) vs. 130.0 (88.6~196.5) on day − 1, *p* = 0.013, 174.2 (92.8~258.1) vs. 174.2 (114.3~263.4) on day 3, *p* = 0.304). Changes in UO and sCr were also greater in the diuretics group (control group versus. diuretics group: changes in UO on day 3, 0.37 (0.08~0.74) vs. 0.52 (0.26~0.86), *p* = 0.003; changes in sCr on day 3, − 0.03 (− 0.37~0.35) vs. 0.30 (− 0.09~0.61), *p* < 0.001). Similarly, the diuretics subgroup of the HD initiation and CRRT re-initiation groups had greater changes in UO and sCr compared to the control subgroup in the same groups.

### Impact of furosemide administration method

In the CRRT discontinuation group, the effects of diuretic administration method on UO and sCr were further analyzed. The average doses of diuretics in each group are summarized in Table 2. Patients in the CIV subgroup received significantly higher doses of furosemide compared to those in the INT subgroup. The furosemide CIV (high-dose furosemide) subgroup tended toward increased UO compared to other methods. UO was similar among subgroups on day − 1, whereas the CIV subgroup had greater UO compared to other subgroups on day 3. However, the CIV subgroup also had a greater increase in sCr compared to the other two methods when CIV was maintained for more than 1 day (Fig. 2e and f). Changes in UO and sCr were greater in the CIV subgroup compared to the other two methods.

**Table 2:** Average dose of diuretics in each subgroup

Diuretics	Furosemide INT	Furosemide CIV	Furosemide PO	Combination
	*n* = 177	*n* = 73	*n* = 32	*n* = 105
Total furosemide (mg/day)	28.7 ± 41.2	157.9 ± 123.5	32.8 ± 21.4	76.3 ± 103.4
Thiazide (mg/day)	0	0	0	6.3 ± 17.6
Spironolactone (mg/day)	0	0	0	36.3 ± 27.0

Values are mean ± SD (mg/day)

*Abbreviations: CIV* continuous intravenous infusion, *INT* intermittent intravenous administration, *PO* per oral administration

### Hospital mortality and length of ICU and hospital stay

The overall outcomes of each group are summarized in Table 3. Both hospital mortality and ICU mortality were highest in the CRRT re-initiation group. Both the CRRT discontinuation group and the HD initiation group had significantly lower hospital mortality and ICU mortality compared with the CRRT re-initiation group (*p* < 0.001). There was no statistically significant difference in hospital mortality between the CRRT discontinuation group and the HD initiation group (*p* < 0.017), but ICU mortality was significantly lower in the HD initiation group (*p* = 0.002). The length of hospital stay was not significantly different between the three groups, whereas both the CRRT discontinuation group and HD initiation group had a shorter length of ICU stay compared to the CRRT re-initiation group (*p* < 0.001).

**Table 3:** Overall outcomes of each group

Outcomes	CRRT discontinuation group (*n* = 517)	HD initiation group (*n* = 310)	CRRT re-initiation group (*n* = 349)	*p* value
Length of ICU stay (days), median (IQR)	9 (5–19)	7 (4–17)	16 (9–29)	< 0.001^ab^
ICU mortality, *n* (%)	77 (14.9)	23 (7.4)	133 (38.1)	< 0.001^ab^
Length of hospital stay (days), median (IQR)	36 (25–65)	45 (22–72)	41 (24–69)	0.05
Hospital mortality, *n* (%)	163 (31.5%)	73 (23.5%)	198 (56.7%)	< 0.001^ab^

Continuous variables were compared using the Kruskal-Wallis test followed by Mann-Whitney test, and categorical variables were compared using χ^2^ tests

*Abbreviations: ICU* intensive care unit, *IQR* interquartile range

^a^*p* ≤ 0.001 for CRRT discontinuation group versus CRRT re-initiation group

^b^*p* ≤ 0.001 for HD initiation group versus CRRT re-initiation group

### Predictive factors for successful CRRT discontinuation

**Fig. 3:**
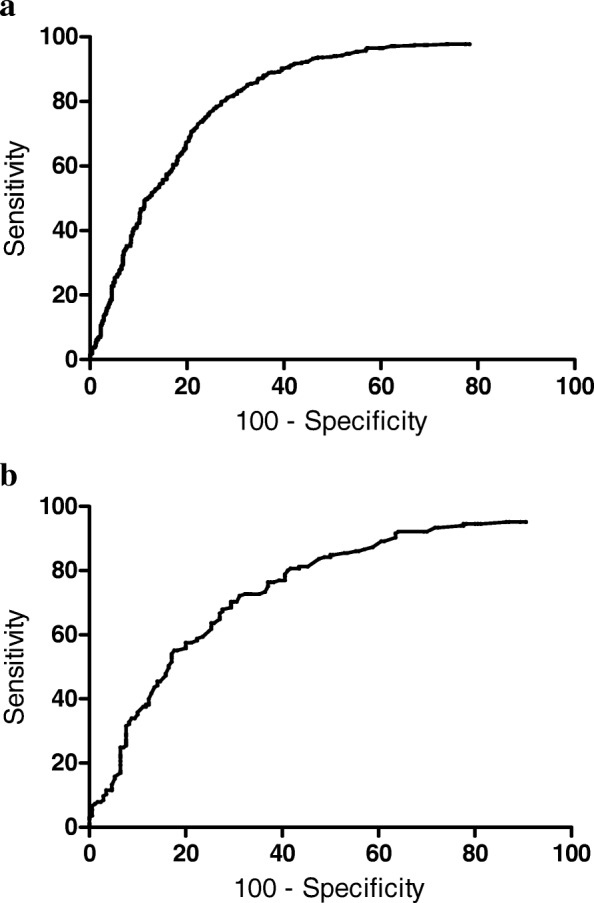
Predictive ability of urine output one day before continuous renal replacement therapy (CRRT) discontinuation [day minus 1 (day − 1)] for discontinuation of CRRT in the diuretics group. **a** Receiver operating characteristic (ROC) analysis of the day − 1 urine output for prediction of successful discontinuation of CRRT. Optimal cutoff value of the day − 1 urine output in all patients was 191 mL/day. The area under the ROC curve was 0.821 (95% CI 0.797–0.845, *p* < 0.001), sensitivity was 81.2% (95% CI 77.6–84.5%), and specificity was 71.6% (95% CI 68.0–75.0%). **b** ROC analysis of the day − 1 urine output for predicting successful discontinuation of CRRT in oliguric (urine output ≤ 400 mL/day) patients in the diuretics group. Optimal cutoff value of the day − 1 urine output was 125 mL/day. The area under the ROC curve was 0.745 (95% CI 0.692–0.798, *p* < 0.001), sensitivity was 72.1% (95% CI 64.6–78.8%), and specificity was 68.8% (95% CI 61.3–75.7%)

Multivariable logistic regression analysis was performed using age, mean blood pressure on day − 1 and day 0, UO on day − 1, total duration of CRRT, use of diuretics after stopping CRRT, vasopressor requirement after stopping CRRT, and several comorbid conditions such as diabetes mellitus, hypertension, and heart failure. Overall, high UO on day − 1, use of diuretics, and short duration of CRRT were predictive factors for successful discontinuation of CRRT (Table 4). ROC analysis of the day − 1 UO for prediction of successful discontinuation of CRRT was conducted (Fig. 3a). The optimal cutoff value of day − 1 UO was 191 mL/day, corresponding to an area under the ROC curve of 0.821 (95% CI 0.797–0.845, *p* < 0.001).

**Table 4:** Predictive factors for successful discontinuation of continuous renal replacement therapy

	Univariable analysis		Multivariable analysis			
	β coefficient	*p* value	β coefficient	Odds Ratio	95% CI	*p* value
Age	0.001	0.789				
Day −1 urine output	0.001	< 0.001	0.001	1.001	1.001–1.002	< 0.001
Duration of CRRT	− 0.084	< 0.001	−0.085	0.919	0.878–0.961	< 0.001
Diuretics	1.726	<.001	1.710	5.529	4.120–7.410	< 0.001
Day −1 MBP	0.000	0.957				
Day 0 MBP	0.000	0.897				
Vasopressors	−0.097	0.538				
Hypertension	−0.142	0.391				
Diabetes mellitus	0.152	0.404				
Heart failure	−0.175	0.637				

Values are based on univariable and multivariable logistic regression models with stepwise selection

*Abbreviations: CI* confidence interval, *CRRT* continuous renal replacement therapy, *Day 0* day of CRRT discontinuation, *Day − 1* one day before CRRT discontinuation, *MBP* mean blood pressure

In patients who were treated with diuretics, high day − 1 UO and short duration of CRRT were predictive factors for diuretic effectiveness, which was defined as successful discontinuation of CRRT and > 400 mL/day of UO after stopping CRRT. ROC analysis of the day − 1 UO for predicting successful discontinuation of CRRT in oliguric patients (UO ≤ 400 mL/day before stopping CRRT) in the diuretics group revealed that the optimal cutoff value of day − 1 UO was 125 mL/day, with an area under the ROC curve of 0.745 (95% CI 0.692–0.798, *p* < 0.001) (Fig. 3b).

## Discussion

In this study, we demonstrated that starting diuretics at the cessation of CRRT contributes to successful discontinuation of CRRT by reducing volume overload risk in critically ill patients with AKI. Patients treated with diuretics following CRRT had a greater increase in UO, with tolerable elevation of sCr not only in the CRRT discontinuation group, but also in other groups. Continuous infusion of furosemide increased UO more effectively compared to other methods. High day − 1 UO, use of diuretics, and short duration of CRRT were predictors of successful discontinuation of CRRT. The cutoff values for day − 1 UO for predicting successful discontinuation of CRRT in all patients and oliguric patients in the diuretics group were 191 mL/day and 125 mL/day, respectively.

Most patients who survive AKI requiring CRRT are reported to recover from oliguria and uremic complications after 12–13 days of RRT [20, 21]. Insufficient urine volume is a common problem in critically ill patients with AKI when discontinuation of CRRT is under consideration. Re-initiation of CRRT is required in a substantial portion of patients with incomplete renal recovery [22]. Previous studies have reported that furosemide does not improve renal outcome after RRT [11, 15]. Uchino et al. reported an association between diuretic therapy within 24 h before stopping CRRT and successful discontinuation of CRRT [12]. However, no previous reports have focused on the clinical impact of diuretic therapy initiated after stopping CRRT on successful discontinuation of CRRT and optimal indications or appropriate administration methods for diuretics following CRRT, even though a positive fluid balance is associated with delayed recovery of kidney function and increased mortality [23, 24]. Our large cohort study demonstrated that diuretics were significantly associated with successful discontinuation of CRRT, and continuous infusion of furosemide was more effective at increasing UO than other methods of administering diuretics. Diuretic therapy was even effective in oliguric patients with UO greater than 125 mL/day before stopping CRRT.

There is still no consensus on the management of patients with AKI discontinuing CRRT. When to discontinue CRRT in patients with AKI depends on various factors [25, 26]. A previous study reported that the possibility of successful CRRT discontinuation is higher in patients with a relatively greater baseline UO or no history of chronic kidney disease [12]. A high 6-h UO immediately before CRRT initiation is associated with lower mortality [27]. Similarly, patients in the CRRT discontinuation group had higher UO compared to patients resuming RRT at both initiation and cessation of CRRT in our study. The HD initiation group had higher sCr and had a greater proportion of hypertensive patients compared with other groups. On the other hand, patients with diuretics in the HD initiation group also had increased UO. Therefore, diuretics may reduce the risk of volume overload regardless of re-initiation of RRT.

In our study, patients treated with diuretics had significantly greater increase in UO, although sCr elevation also occurred. The clinical impact of diuretics on the outcome of AKI has been evaluated in several studies. A previous systematic review reported that furosemide has no significant effect on renal outcome, including RRT and in-hospital mortality [28]. One randomized placebo-controlled study showed that furosemide significantly increased urine volume but did not facilitate post-RRT renal recovery [15]. The best method for evaluation of actual renal function during the peri-RRT period remains unclear [29], although continuous solute clearance by CRRT is known to stabilize sCr after 48 h [30]. Other observational studies have reported UO as the most significant predictor of successful termination of CRRT [11, 12]. Furthermore, UO during the first 2 h after a furosemide stress test shows predictive value for prognosis of AKI [31] and creatinine clearance > 30 mL/h assessed by 6-h urine collection has been suggested as a criterion for discontinuing RRT [32]. Therefore, both our study and several previous reports support the clinical importance of UO as a better indicator of actual renal function than sCr in patients with AKI who discontinued CRRT.

Regarding the methods of administering diuretics, there has been only one study comparing torasemide and furosemide following CRRT in patients with AKI after cardiac surgery. Both loop diuretics were effective for diuresis, while torasemide showed better dose response effects than furosemide [33]. Our study analyzed more diverse administration methods, including combination treatment, and showed that continuous infusion of furosemide was the most effective in increasing UO. These results suggest that continuous infusion of furosemide would be beneficial for patients with AKI discontinuing CRRT, especially those who are at risk of volume overload, because fluid overload is a major complication of AKI [34]. High UO induced by furosemide might protect the injured kidneys from necrotic debris and sludge blocking the tubular lumen through increased tubular flow [35].

Multivariable regression analysis revealed greater UO on day − 1, shorter duration of CRRT, and use of diuretics as predictive factors for successful discontinuation of CRRT. UO before stopping CRRT has previously been reported as a significant predictive factor for successful discontinuation of RRT [11–13, 27]. The 2-h UO after a furosemide stress test has been suggested as a novel prognostic marker for RRT in AKI compared with several urinary biomarkers [36]. Shorter duration of CRRT has also been reported as a predictive factor for renal recovery in previous studies [11–13]. Despite the utility of RRT in severe AKI, timely discontinuation of RRT needs to be attempted as RRT itself might delay renal recovery through hemodynamic instability and catheter-related infection [37]. Although diuretics were expected to be beneficial in patients with AKI discontinuing CRRT due to increased UO, the evidence is lacking. Our finding that diuretics are a predictive factor for successful discontinuation of CRRT is clinically meaningful because most previous studies have failed to prove a beneficial effect of diuretics following CRRT, except for one small study by Heise [11–13, 15]. In addition, 191 mL/day of UO before stopping CRRT was found to be the optimal cutoff value for predicting successful discontinuation of CRRT in our study, while a previous study reported a UO cutoff value of 436 mL/day [12]. Furthermore, we found that 125 mL/day UO on day − 1 was the optimal cutoff value for predicting successful discontinuation of CRRT by diuretic therapy in oliguric patients. The urine volume cutoff for diuretic therapy in the CRRT discontinuation group was smaller in our study compared to the previous report [12]. The difference between their report and our study in the prediction of urine volume for successful discontinuation of CRRT may be attributed to the difference in the study population and the time period of successful discontinuation of CRRT: 7 days in their study versus 3 days in our study. We believe that differences in regional or institutional policies regarding operation protocols, restart indications, or the insurance reimbursement system for CRRT may also contribute to the difference in urine volume cutoff.

Several limitations in our study deserve consideration. First, the study was a retrospective cohort study of both medical and surgical ICU patients. Multivariable analyses adjusted with patient comorbidities were conducted to minimize the impact. In particular, blood pressure and vasopressor requirements were also adjusted. Only a few small studies have investigated the predictive factors for restoration of renal function after discontinuation of CCRT [11–13]. Our study showed the clinical potential of diuretics in patients with AKI discontinuing CRRT and suggested clinical significance based on a large cohort. However, the degree of diuresis before attempting CRRT discontinuation was still a confounding factor affecting both initiation and maintenance of diuretic therapy in our cohort. To overcome this inevitable critical limitation, we performed a multivariable analysis including UO on the day before CRRT discontinuation, and diuretic therapy was identified as a significant predictor of successful discontinuation of CRRT. A well-designed, randomized, controlled trial is required to further clarify the clinical effectiveness of diuretics. Second, long-term outcome was not analyzed. Since various confounding events can occur in critically ill patients with AKI and the main purpose of this study was to investigate the clinical effectiveness of diuretics for successful discontinuation of CRRT, we focused on the short-term renal outcome up to 3 days after stopping CRRT and briefly investigated short-term overall outcomes. Patients who died within 3 days following discontinuation of CRRT were excluded from the analyses to reduce confounding factors for evaluating the clinical usefulness of diuretics in patients with AKI who are discontinuing CRRT. Third, in previous studies, fractional excretion of urea and furosemide stress test were studied as predictors of AKI prognosis [31, 38] and creatinine clearance assessed by 6-h urine collection was suggested as a predictor of successful CRRT discontinuation [32]. However, these parameters could not be included in our study because of the retrospective study design.

## Conclusions

In conclusion, diuretic therapy following CRRT increased UO significantly with tolerable elevation of sCr. Day − 1 UO, use of diuretics, and duration of CRRT were significantly related to successful CRRT discontinuation. Aggressive use of diuretics seemed more helpful in oliguric patients whose UO was ≥ 125 mL/day on the day before stopping CRRT. Our study supports the clinical usefulness of diuretic therapy in critically ill patients with AKI by inducing favorable fluid balance, especially those who are at risk of volume overload after discontinuation of CRRT.

## Abbreviations

AKI: Acute kidney injury
BUN: Blood urea nitrogen
CI: Confidence interval
CIV: Continuous intravenous infusion
CRRT: Continuous renal replacement therapy
Day − 1: One day before continuous renal replacement therapy discontinuation
INT: Intermittent intravenous administration
IQI: Interquartile interval
KDIGO: Kidney disease: improving global outcomes
MDRD: Modification of diet in renal disease
PO: Per oral administration
ROC: Receiver operating characteristic
RRT: Renal replacement therapy
sCr: Serum creatinine
SD: Standard deviations
UO: Urine output
